# Supplementary material for: Neural network kinetics for exploring diffusion multiplicity and chemical ordering in compositionally complex materials
Source: Nat Commun. 2024 May 9;15:3879. doi: 10.1038/s41467-024-47927-9 (PMC11082203; doi:10.1038/s41467-024-47927-9)
Supplement: Supplementary file 1 — Supplementary Information [file 41467_2024_47927_MOESM1_ESM.pdf]

Supplementary Information

Neural Network Kinetics for Exploring Diffusion Multiplicity and Chemical Ordering in Compositionally Complex Materials

Table of Contents

Supplementary Figures 1-9	2
Supplementary Note 1: On-lattice structure and chemistry representation	11
Supplementary Note 2: Determining the cutoff distance	14
Supplementary Note 3: Architecture of neural network and convolutional neural network	16
Supplementary Note 4: Number of compositions for predicting the entire ternary composition space	20
Supplementary Note 5: Comparison with cluster expansion method	23
Supplementary Tables 2-3	25

Supplementary Figures

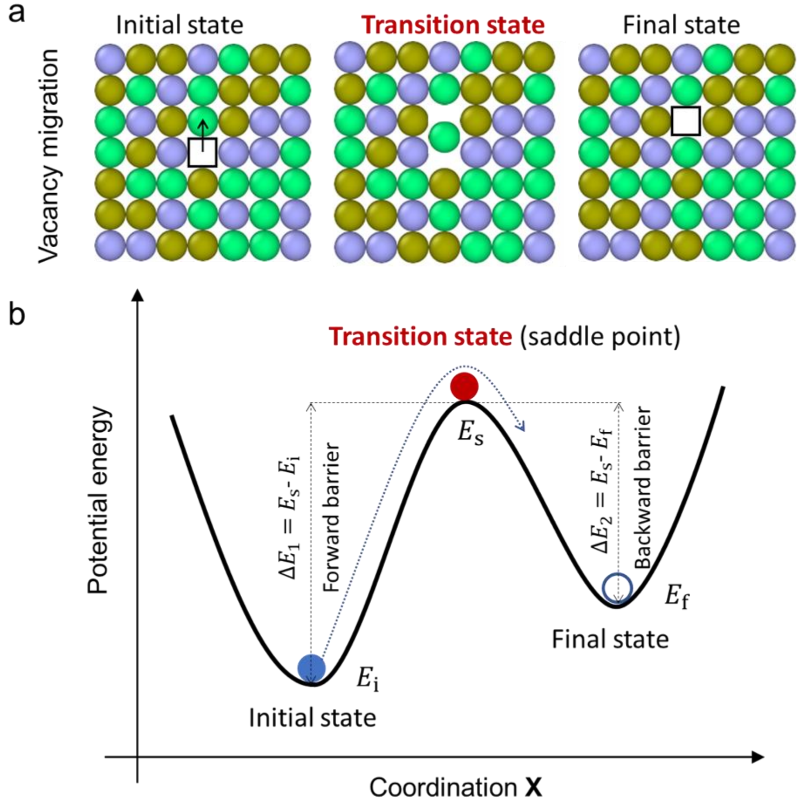

Figure S1. Schematic illustration of vacancy diffusion and the corresponding diffusion energy landscape. (a) Vacancy diffusion states from an initial state, through saddle point, and leads to the final state. (b) The energy barrier ΔE, i.e., the energy difference between transition state and the initial energy minimum, is the governing value for diffusion. The key task is to accurately and efficiently predict these barriers in compositionally complex materials.

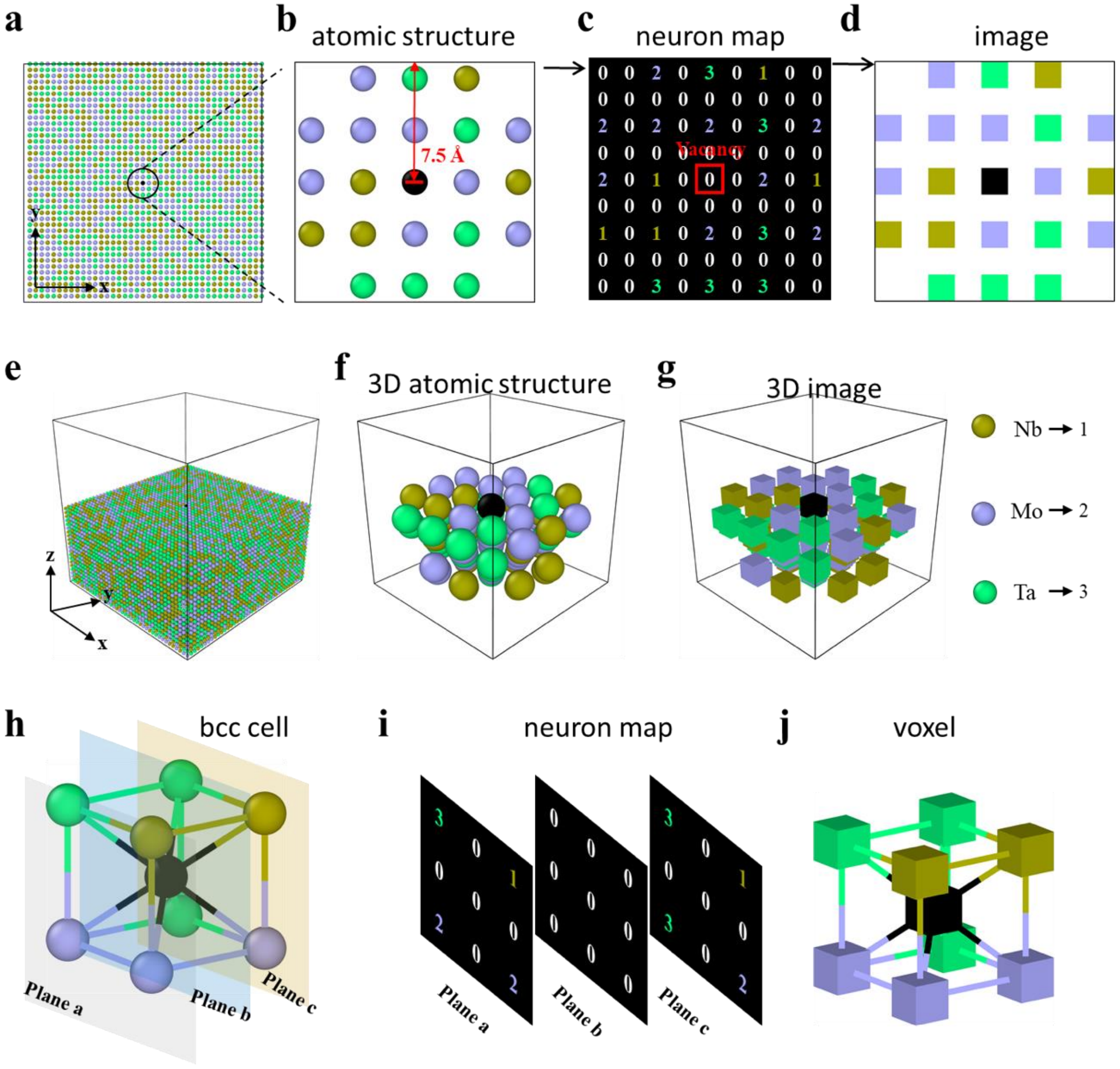

Figure S2. On-lattice presentation of local atomic environments in equimolar NbMoTa alloy. (a) Atom plane containing a vacancy (color-coded by black). (b) Enlarged view of the region within the circle region in (a) with cutoff distance 7.5 angstroms. (c-d) Digit matrix (neuron map) converted from atomic structure. (e-g) 3D illustration of atomic configuration within/below the vacancy-containing layer. (h) Vacancy and its first nearest neighboring atoms, and (i-j) the corresponding neuron map. The nearest neighbors are determined based on Euclidean distance between vacancy and atoms.

Table S1. Operations of aligning eight diffusion pathways with the reference direction.

Path	Rotation	Mirror	Path	Rotation	Mirror
V-1	0	No	V-5	0	Yes
V-2	0.5π	No	V-6	0.5π	Yes
V-3	π	No	V-7	π	Yes
V-4	-0.5π	No	V-8	-0.5π	Yes

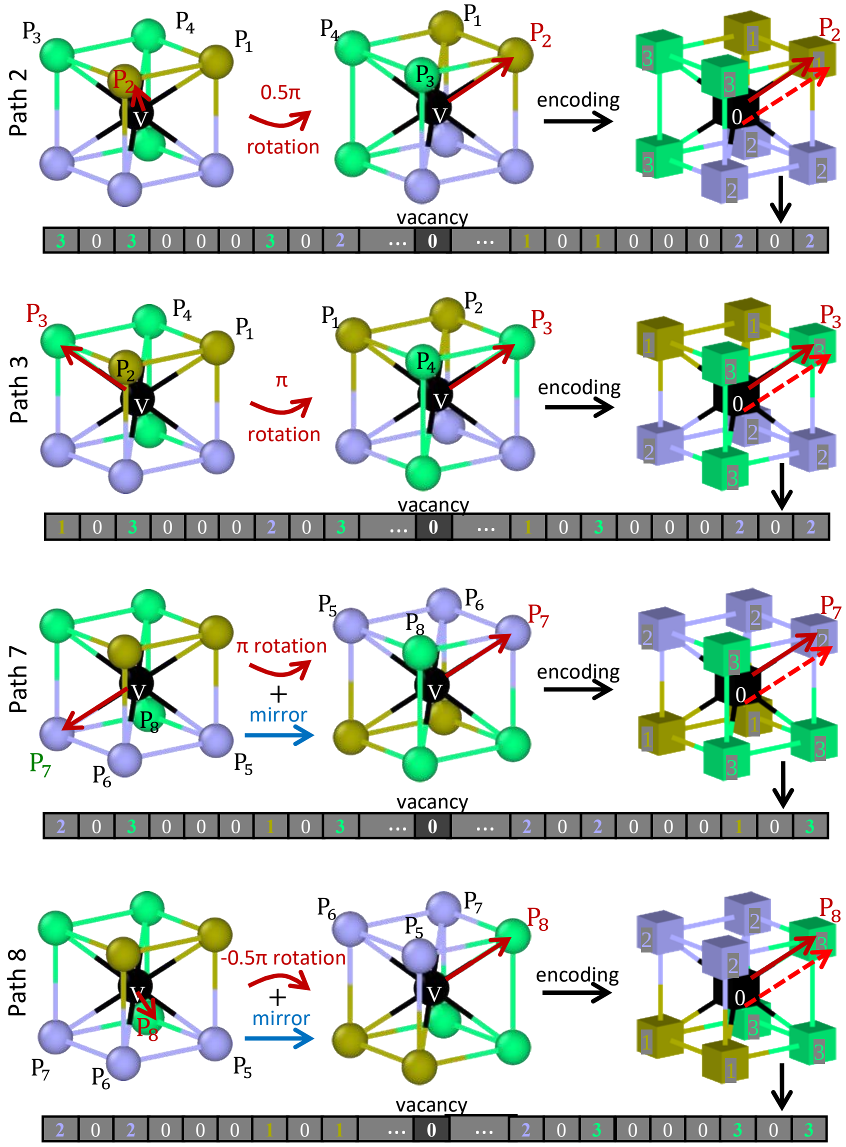

Figure S3. Aligning diffusion pathways 2, 3, 7 and 8 with the reference direction. The symbol V represents the vacancy.

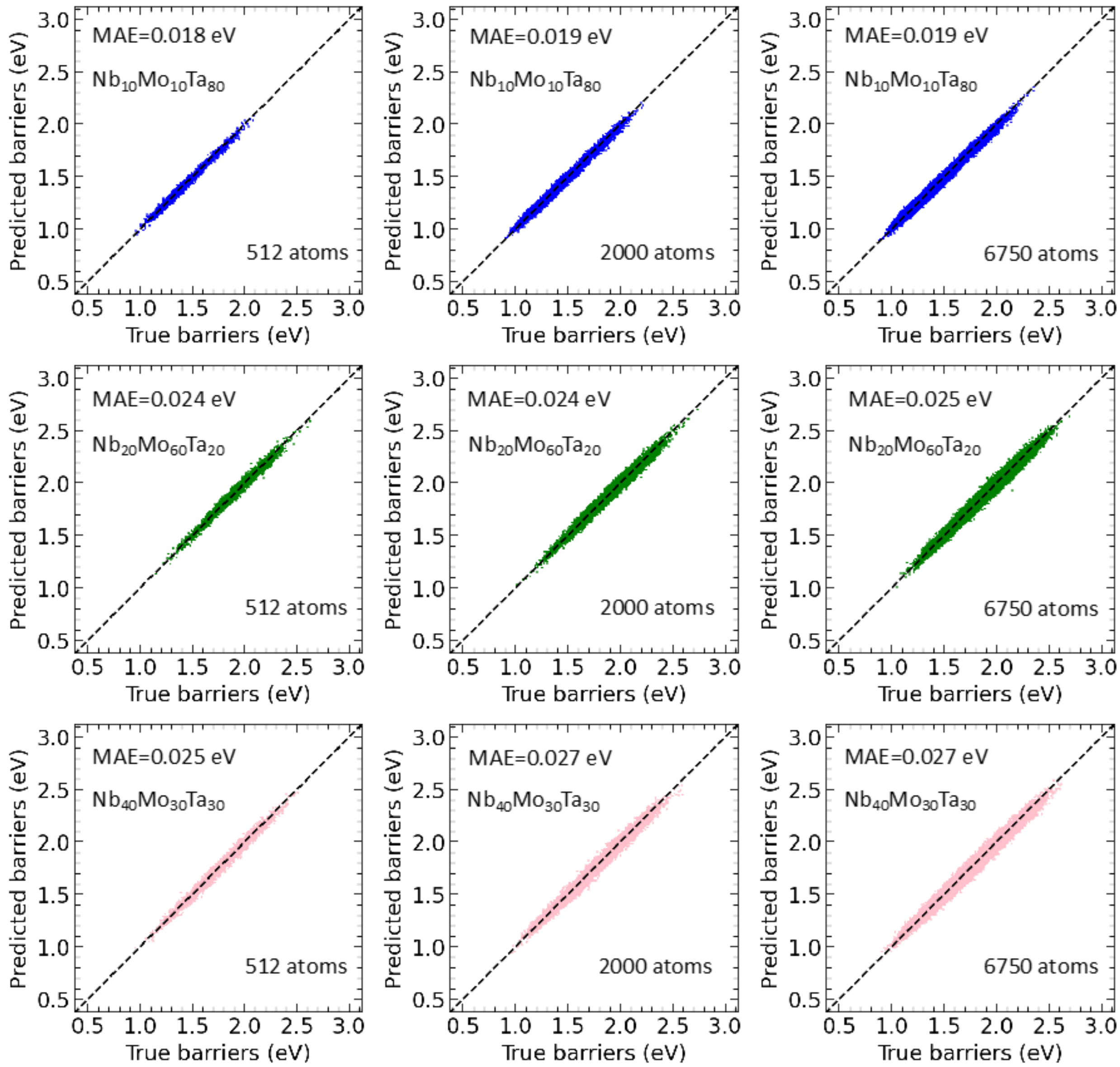

Figure S4. Performance of neural network in predicting diffusion barrier spectrum in unseen compositions and varying system sizes (scalability). Three compositions, including $Nb_{10}Mo_{10}Ta_{80}$, $Nb_{20}Mo_{60}Ta_{20}$, $Nb_{40}Mo_{30}Ta_{30}$, and three systems containing 512, 2000, and 6750 atoms are shown.

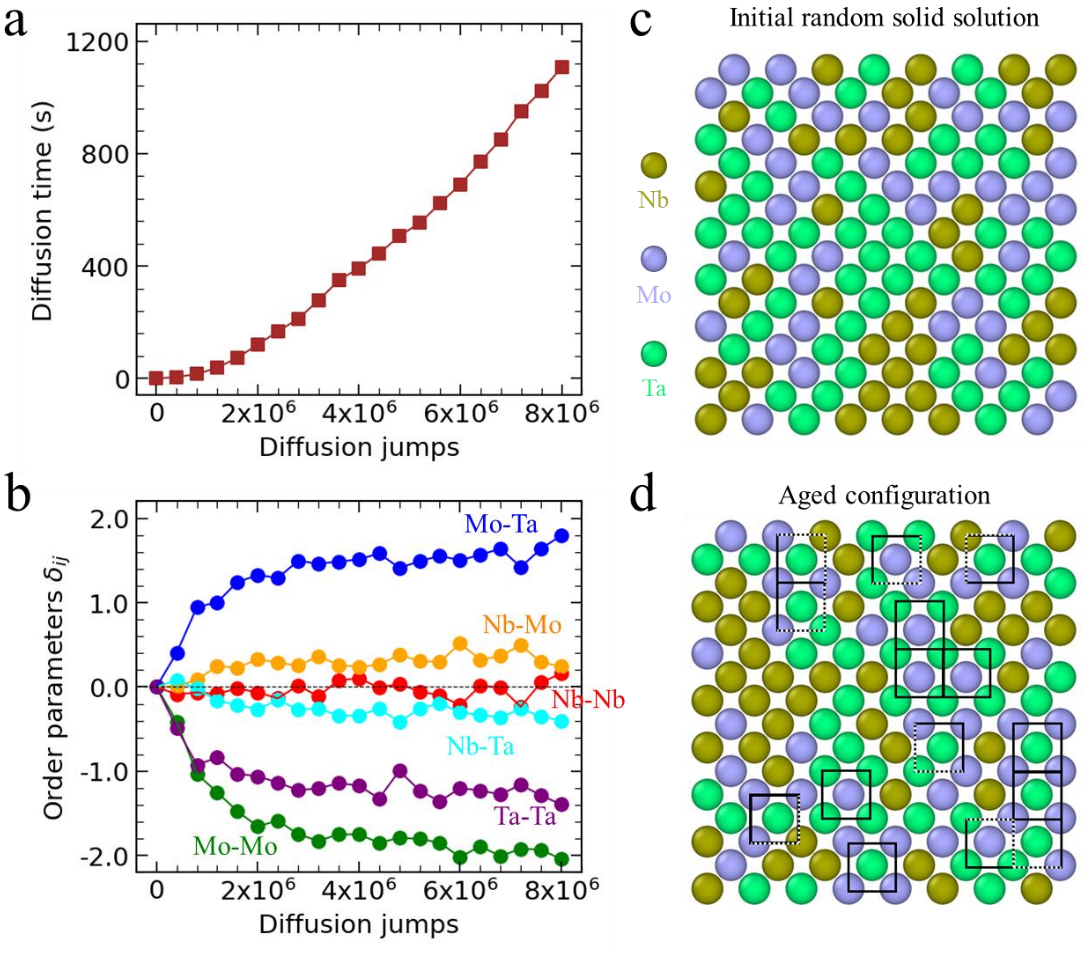

Figure S5. Diffusion and chemical ordering in NbMoTa alloy from NNK simulation at 1,000 K. (a) The accumulated diffusion time as a function of jumps. (b) Variation of chemical order parameters with jump. (c) Initial atomic configuration with random solid solution, and (d) aged structure demonstrating B2 ordered cluster.

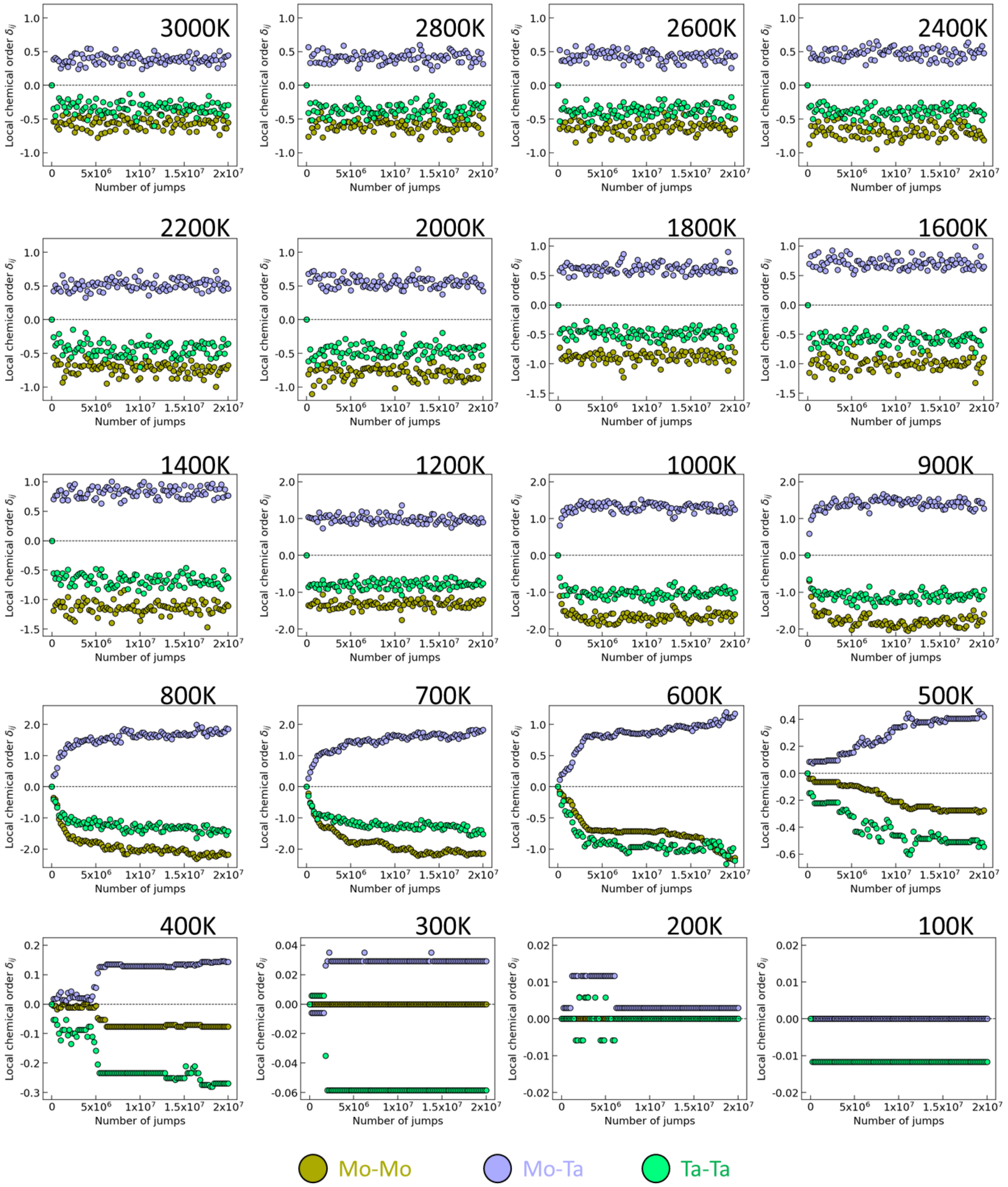

Figure S6. Variation of the chemical order parameter as a function of diffusion jump obtained from NNK simulation. The simulations are conducted at twenty different temperatures, ranging from 3,000 K to 100 K, as indicated in the labels.

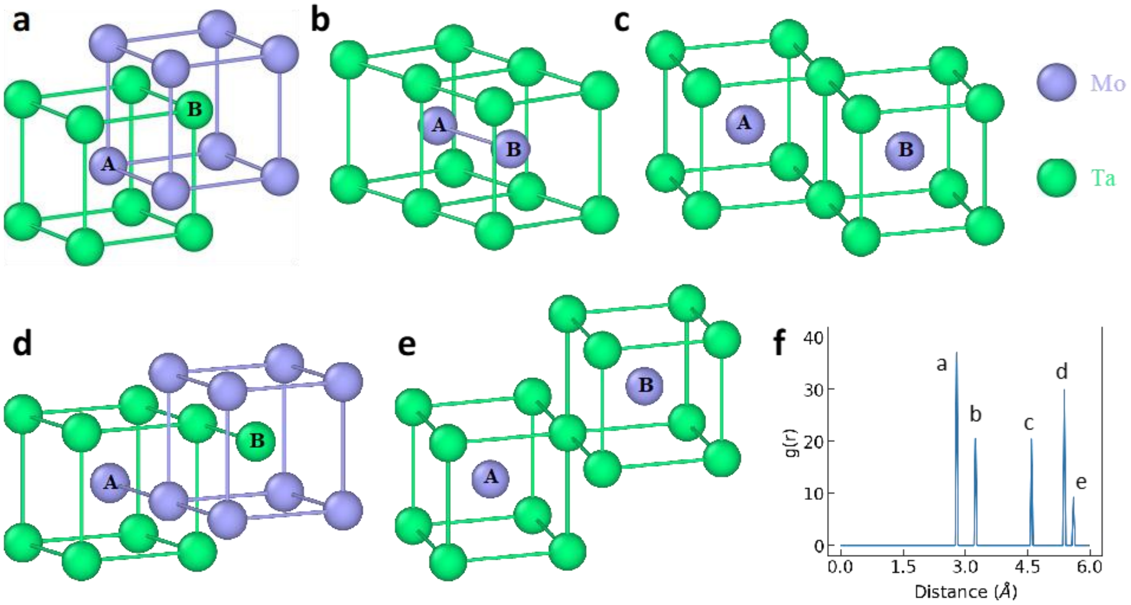

Figure S7. B2 cluster identification. (a-e) A cluster consists of two B2 cells that share volume, face, edge, and vertices. (f) The corresponding separation distance between the two B2 cells.

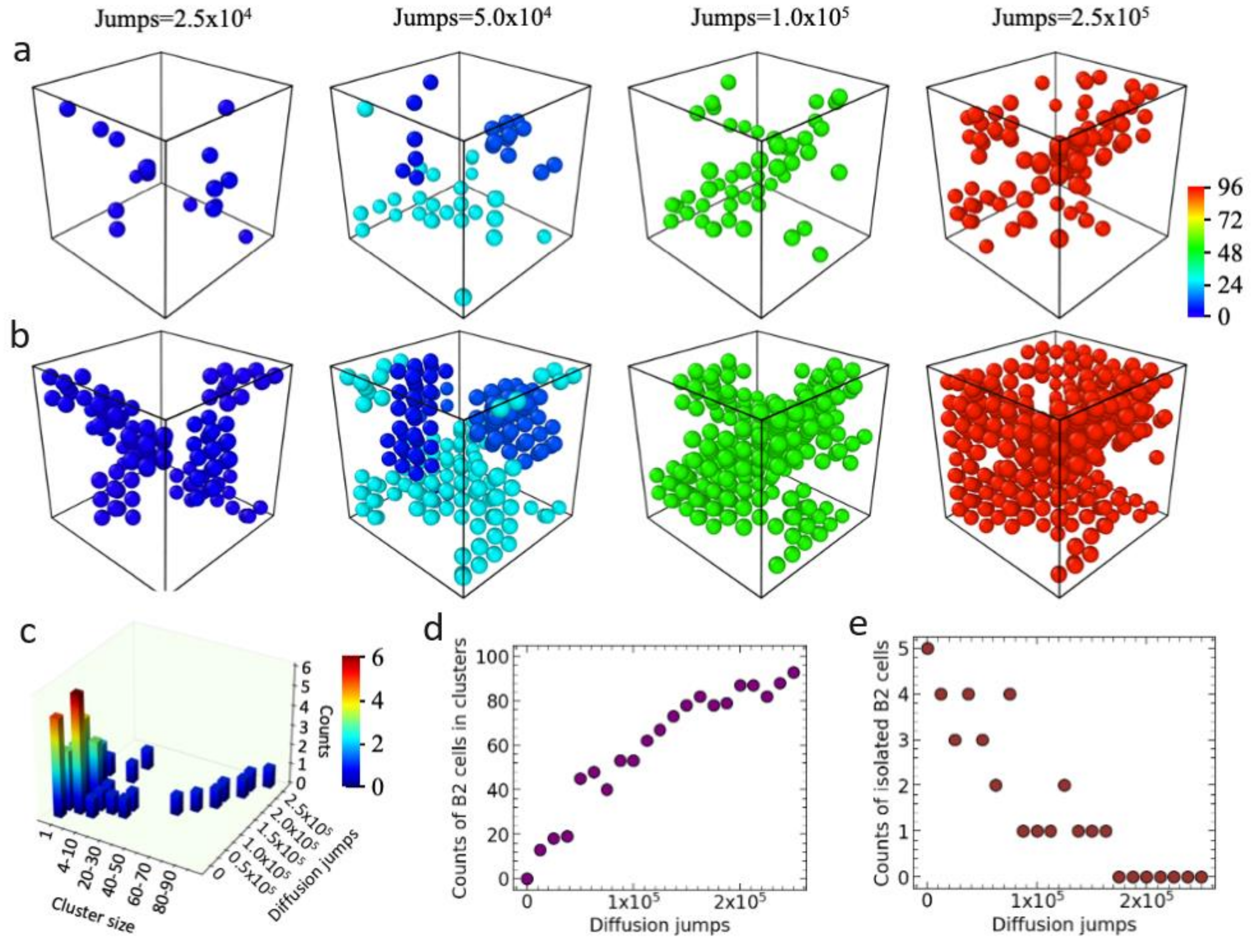

Figure S8. Formation and coalescence of B2 clusters in a small equimolar NbMoTa model. Panel. (a) shows the variation in B2-centred atoms as the number of jumps increases. (b) the same configuration is displayed, but with the entire B2 cells visible. After 10^5 jumps, the two clusters combine into one, represented in green. (c) displays the B2 cluster size distribution obtained after varying numbers of jumps. (d) depicts the number of B2 cells as a function of atomic jumps, with panel (e) indicating a decrease in the number of isolated B2 cells with increasing atomic jumps.

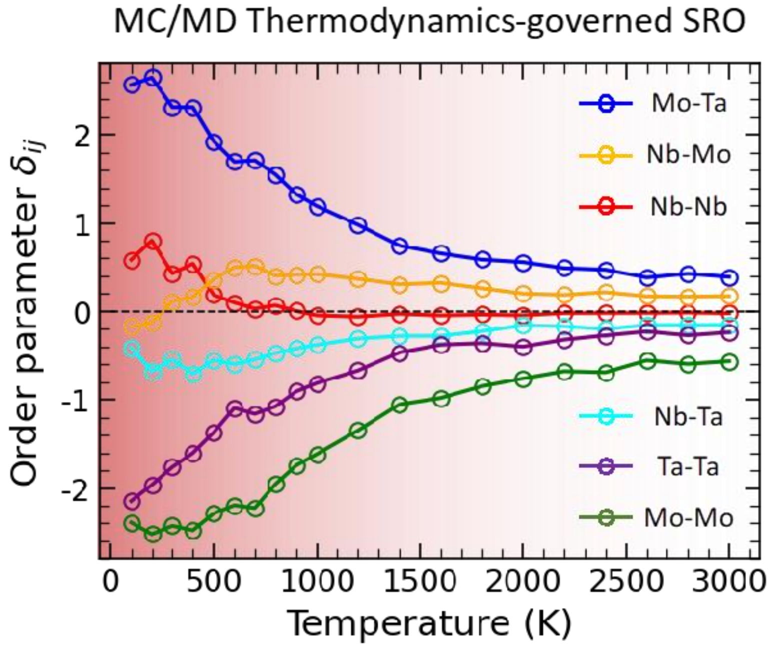

Figure S9. Variation of chemical order obtained at different annealing temperatures using static Monte Carlo with random swap. Variation of chemical order δ_{ij} obtained at different temperatures. The chemical order shows monotonic increases with decreasing temperature.

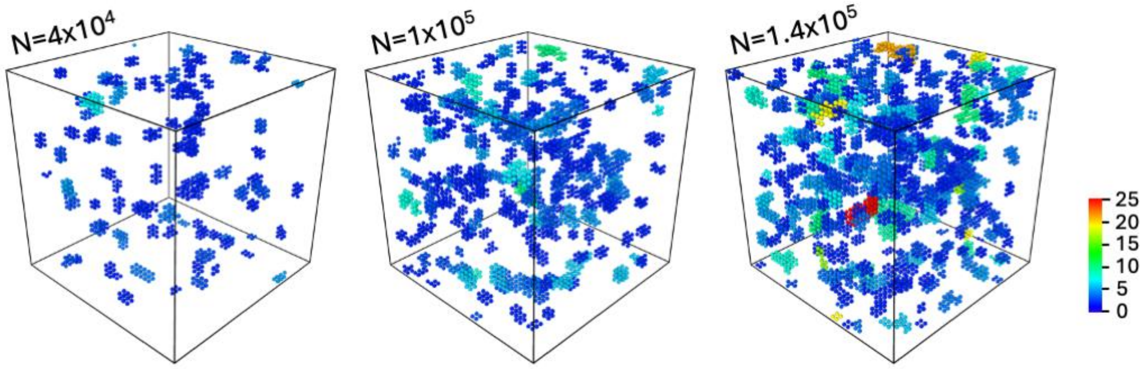

Figure S10. B2 structure morphology generated from a random swap MCMD simulation, exhibiting a more uniform distribution. Spatial distributions of growing B2 clusters with the number of MC swaps N.

Supplementary Note 1: On-lattice structure and chemistry representation

We use on-lattice representation to convert local atomic environments into digital matrices in which each value represents one atom or vacancy. To achieve this, we follow two rules: divide the material model into a grid of pixels; place each atom at the center of one pixel. With the periodicity of crystalline structures, the rules provide us guidance in digitalizing the material model reasonably.

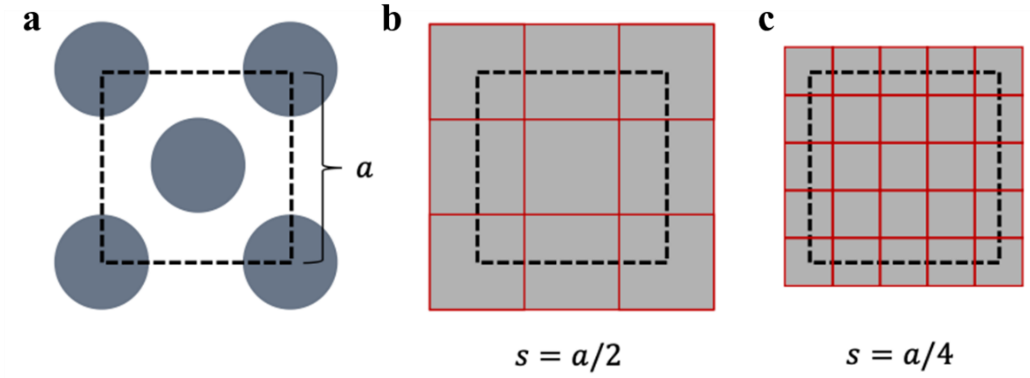

Figure S11. On-lattice representation and pixel size determination. (a) A unit cell with lattice constant a. (b-c) depicts the grid separating the atomic model into uniform cells (pixels), with the pixel size $s = a/2$ (b) and $s = a/4$ (c).

The Figure S11 schematically illustrates the on-lattice representation, which converts a 2D atomic structure into a matrix. The conversion is achieved using a pixel grid, which divides the structure into uniform cells or pixels. For bcc structure, the largest grid we can use, which can fully distinct all lattices and yield the smallest voxel grid dimensionality, is $s = a/2$, where a is the lattice constant of the crystal, as shown in Figure S11b. In general, the structure domain can be equally divided into pixels with size $s = a/2n$, where $n = 1, 2\ 3 \dots$. For instance, Figure S11c shows the representation using pixel size $s = a/4$. Once converting the model into pixels, we can encode each pixel based on the local atom type as illustrated in the main text. The selection of pixel size depends on the material structure alone without involving any hyperparameters which typically exist in other structure descriptors. This avoids the need to adjust and select any hyperparameters. Furthermore, it enables us to use the largest pixel that fully captures the local structure and chemical information, reducing the burden of storage and accelerating the training of machine learning models. In Figure S1 (on page 2), we illustrate the process of converting local atomic environments into digit matrices for a 3D crystal.

Rotational non-invariance of neural map (digital matrix). For a given atomic configuration that includes a vacancy, there are eight migration paths associated with the vacancy in bcc crystal. The key challenge lies in how to predict these distinct migration barriers from one neural map

(atomic configuration). To address this, we introduce a 'reference direction', which aims to mark the diffusion path of interest. By performing rotation and mirroring operations on the atomic configuration, we can align the diffusion direction of interest with this reference. Hence, unique digital matrices and digital vectors can be generated for each individual diffusion paths, preserving structural symmetry. Figure S12 below exemplifies this process, showing how diffusion paths 2 and 3 are aligned with the reference direction (indicted by red arrow). The Figure 2 of manuscript and Figure S3 details these operations and the resultant matrices for all diffusion directions.

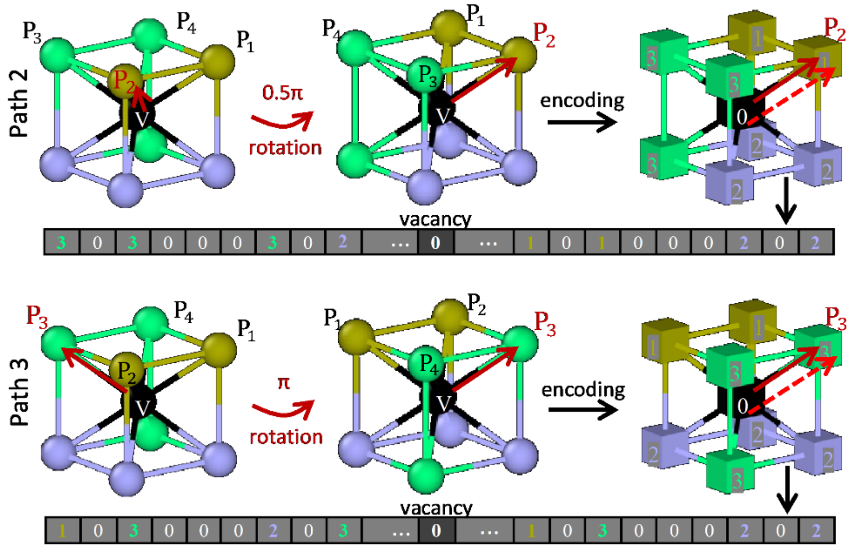

Figure S12. Aligning diffusion paths 2 and 3 with the reference direction through rotation. It produces two digital matrix and vectors corresponding to the two paths.

This rotational non-invariant feature of digital matrix can also be understood from the handwritten digit recognition. For instance, when the MNIST database's handwritten '6' is rotated by 180 degrees, it resembles a '9', as shown in Figure S13. Despite the pixel values in the matrices being unchanged, the orientation relative to the reference direction (denoted by the arrow) allows for the correct interpretation.

The neural network model discerns the overall sequence and pattern in the digital matrix, not individual zeros. In a perfect crystal structure (bcc here), the digital matrix displays a consistent sequence of non-zero and zero digits. However, the introduction of a vacancy alters this structure by adding an additional zero at the corresponding location. This alteration in the digital sequence is what the neural network is trained to detect and learn from, enabling it to predict associated properties.

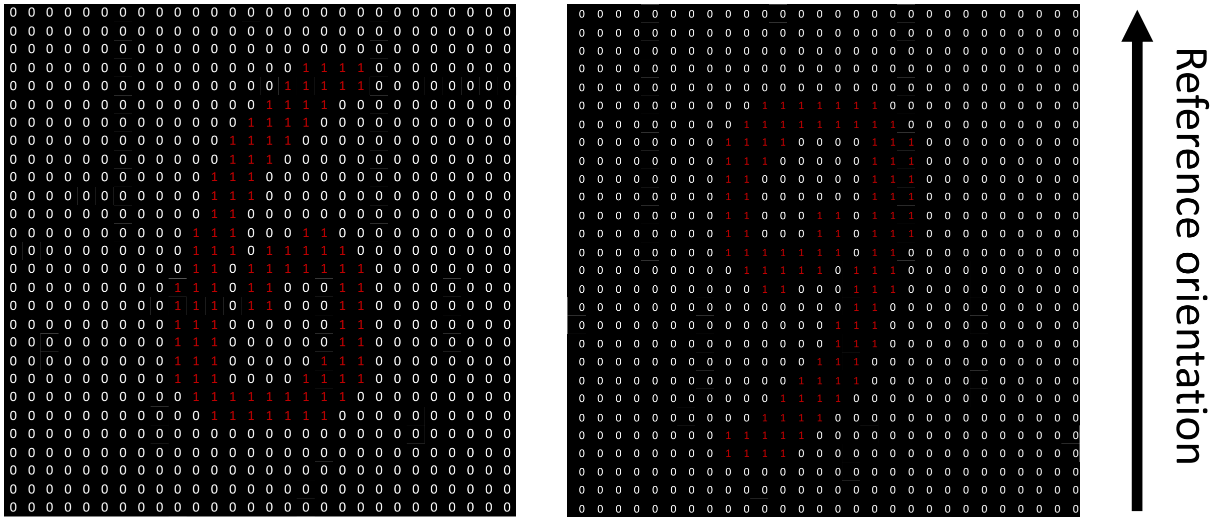

Figure S13. Rotational non-invariance for handwritten digit recognition. The image on left shows the pixel map of digit 6. On the right, the same pixel map has been rotated 180 degrees, indicating the digit 9.

Supplementary Note 2: Determining the cutoff distance

Vacancy diffusion and associated activation barrier depend on local atomic environment. The impact of surrounding atoms on vacancy diffusion should decay as the distance increases. Beyond a certain critical distance, the impact becomes negligible. To determine the critical distance, we examine the dependence of model prediction performance on the cutoff distance. Figure S14a presents a radial distribution function g(r) from a NbMoTa alloy, which indicates that atoms within 7.5 Å are separated into eight shells. When using a larger cutoff distance, we consider atoms in higher order shells, thus more atoms. Figure S14b shows the dependence of number of atoms on the cutoff distance. The number of atoms increases from 8 to 112 when the cutoff distance increases from 3.0 to 7.5 Å (meaning we consider atoms in more shells), leading to a more informative local environment representation.

For each cutoff distance, we create a dataset from four alloys, including $Nb_{33}Mo_{33}Ta_{33}$, $Nb_{50}Mo_{25}Ta_{25}$, $Nb_{25}Mo_{50}Ta_{25}$ and $Nb_{25}Mo_{25}Ta_{50}$. For each composition, we simulate atomic configurations comprising 2,000 atoms (i.e., lattice sites). Considering that each vacancy can migrate in one of eight possible directions, this results in 16,000 unique migration barriers per composition (2,000 vacancies $\times$ 8 directions). Consequently, by studying four distinct compositions, we determine a total of 64,000 barriers (16,000 barriers per composition $\times$ 4 compositions). Table S2 summarizes all the dataset. The dataset is split into two parts, with 80% used for training and 20% for validation. For each cutoff distance, we train a neural network with 4 hidden layers and 128 hidden layer units. Figure S14c shows the mean absolute errors (MAEs) of prediction on both training and validation datasets at different cutoff distances. The validation error decreases from 0.117 eV to 0.036 eV as the cutoff distance increases from 3.0 to 7.5 Å. It almost converges at later stage from 7.0 to 7.5 Å, indicating that 7.5 Å is an effective cutoff distance for representing the local atomic environment. However, we note that the neural network model and dataset have not reach a good balance for most cutoff distances, as evidenced by the gap between training and validation error. Further tuning of the network architecture can solve this problem. Nonetheless, our goal here is solely to demonstrate how the cutoff distance influences the diffusion barrier prediction using identical neural network model for all cases. We expect the conclusion will not change if we further adjust the neural network models at different cases.

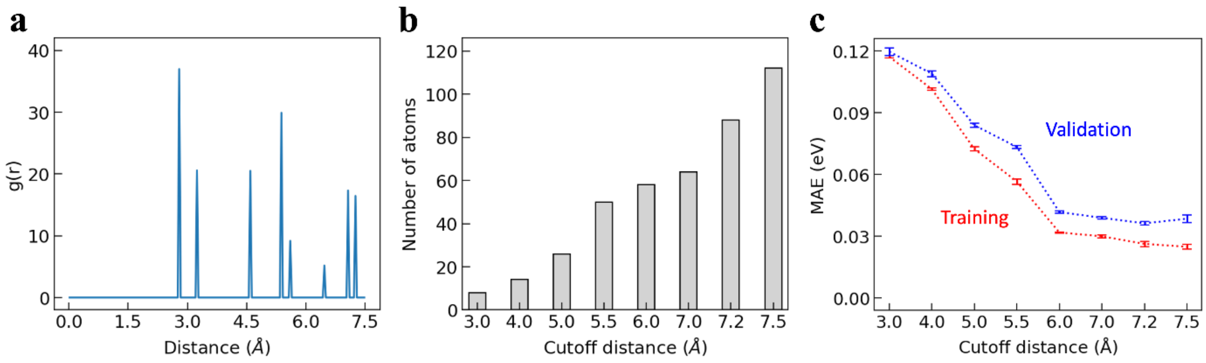

Figure S14. Effect of cutoff distance on neural network prediction. (a) The radial distribution function g(r) of bcc NbMoTa. (b) The number of neighboring atoms surrounding a vacancy as a function of cutoff distance. (c) The machine learning prediction error as a function of cutoff distance has converged at 7.5 Å. The red and blue curve represents the training and validation mean absolute error (MAE), respectively. The error bars represent the standard deviations of model prediction errors using five-fold cross-validation.

Supplementary Note 3: Architecture of neural network and convolutional neural network

The two critical parameters determining the architecture of a neural network include the number of layers, the number of neurons in each layer. To understand the influence of architecture on prediction performance, we train different neural networks using a dataset containing 46 compositions. We compute and generate 736,000 vacancy barriers from these 46 compositions (16,000 barriers from each composition), and the Table S3 summarizes the compositions and dataset. The dataset is split into two parts, 95% as training dataset and 5% as validation dataset. We train a set of neural networks with different numbers of hidden layers (from 1 - 4) and numbers of hidden layer units (16 - 256). We use 69,920 data points (10% of the whole training dataset) to train the networks and then compare the performance of different models on the validation dataset. Figure S15b shows the mean absolute errors of prediction for these models, and Figure S16 presents a direct comparison between true values and predicted value from different neural network models. The prediction error decreases with either increasing the number of layers or neurons and begins to converge for the model with 128 neurons and 2 layers. This suggests that the second order interaction from two hidden layers is sufficient to capture the vacancy-atom interactions. Additionally, the convergence on 128 neurons has physical meaning as they can explicitly capture the 112 neighboring atoms of a vacancy. For modeling vacancy diffusion and B2 ordering in equimolar NbMoTa alloy, the 4-layer neural network model is adopted, owning its robustness in concentrated alloys. It should be noted that, for dilute alloys, a simpler version of the model with 2 layers suffices to accurately predict all barriers and the diffusion-mediated ordering behaviors. In this study on vacancy diffusion and B2 ordering in equimolar NbMoTa alloy, a 4-layer neural network model is used due to its robustness in concentrated solid solutions. For dilute alloys, however, a simpler 2-layer model suffices to accurately predict all barriers and diffusion-mediated ordering behaviors.

In addition to the classic neural network, we have also trained a convolutional neural network (CNN) using the same datasets. Figure S17 depicts the structure of the CNN, which comprises one input layer, four convolutional layers, and one output layer. To the input layer, we feed the 3D neuron map (images), and in each of the four convolutional layers, we apply filters of size $3 \times 3 \times 3$. The number of filters used in the convolutional layers is 32, 64, 128, and 128, which is equivalent to the number of channels of the generated images. Consequently, the data dimension reduces to $1 \times 1 \times 1 \times 128$ from the original $9 \times 9 \times 9 \times 1$. Following each convolutional operation, we apply batch normalization (before the activation function), which provides benefits such as a reduction of sensitivity to model parameter initialization, regularization. The Rectified Linear Unit (ReLU) serves as the activation function, and the data from the final convolutional layer is converted to a one-dimensional vector of length 128 before being passed to the output layer. The output layer, comprising a single neuron, predicts the diffusion barrier. The CNN model is trained for 100 epochs using the Adam optimizer with an initial learning rate of 0.001 and a batch size of 32. After each epoch, the model is evaluated on the validation dataset to monitor the

evolution of the loss, which is represented by the mean square error. If the validation loss fails to decrease after 10 consecutive epochs, the learning rate is decreased by a factor of 10. This smaller learning rate reduces oscillation, avoids divergence of the optimization, and contributes to convergence to the nearby minimum point. The minimum allowed learning rate is 1×10^{-5}, as a learning rate that is too low can greatly slow down the training procedure and waste computational resources. Once the learning rate reaches the minimum value, it remains constant for the rest of the training process. The training procedure ends after 100 epochs, regardless of whether the learning rate has reached the minimum value. The parameters of the model with the best performance are saved for further use.

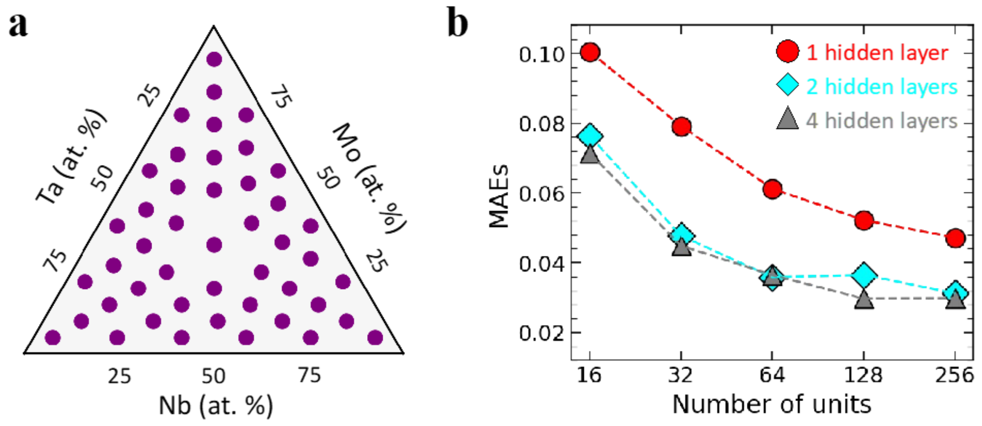

Figure S15. Neural network prediction performance with different numbers of hidden layers and units. (a) For the neural network model, 46 compositions selected from the Nb-Mo-Ta compositional space for training. (b) Different neural network models are evaluated based on their prediction errors on the validation dataset.

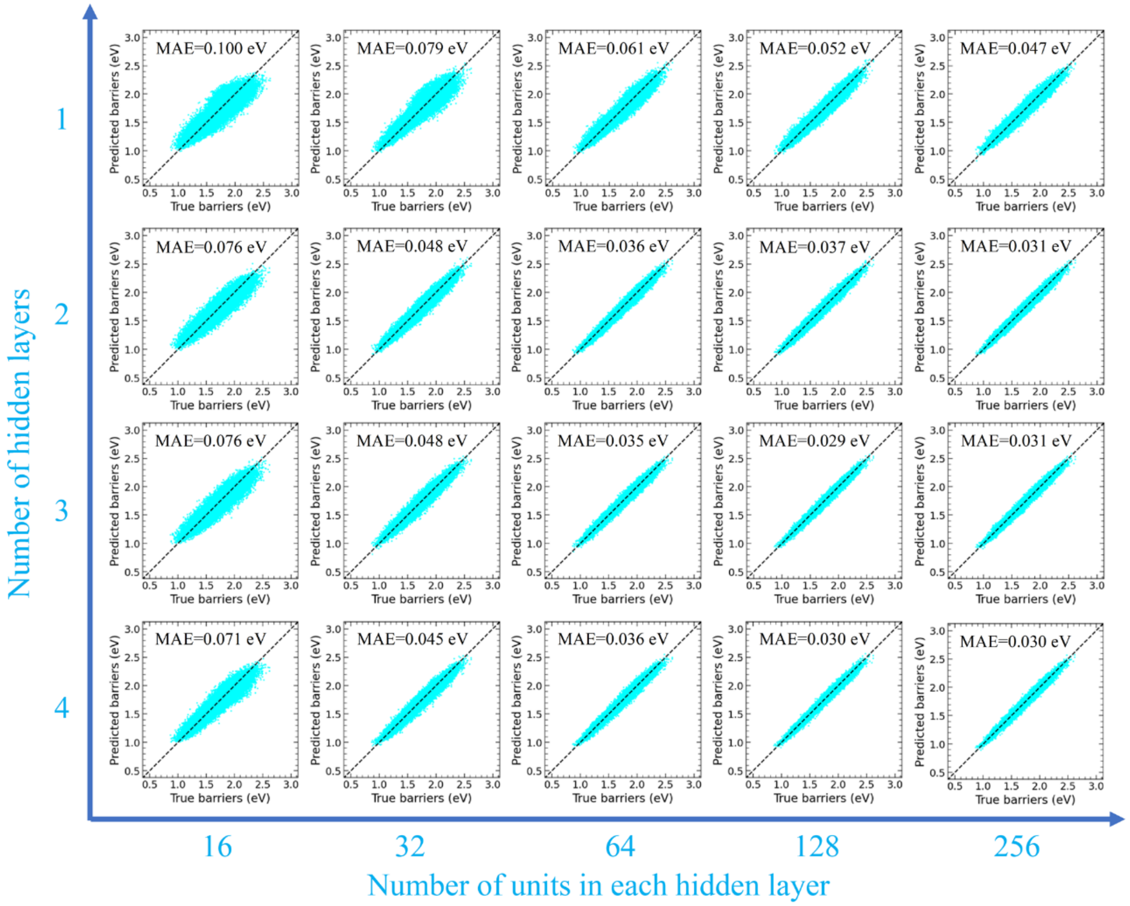

Figure S16. Prediction performance of neural network models with varying hidden layers and the number of units. The prediction accuracy increases with increasing the number of hidden layers and units.

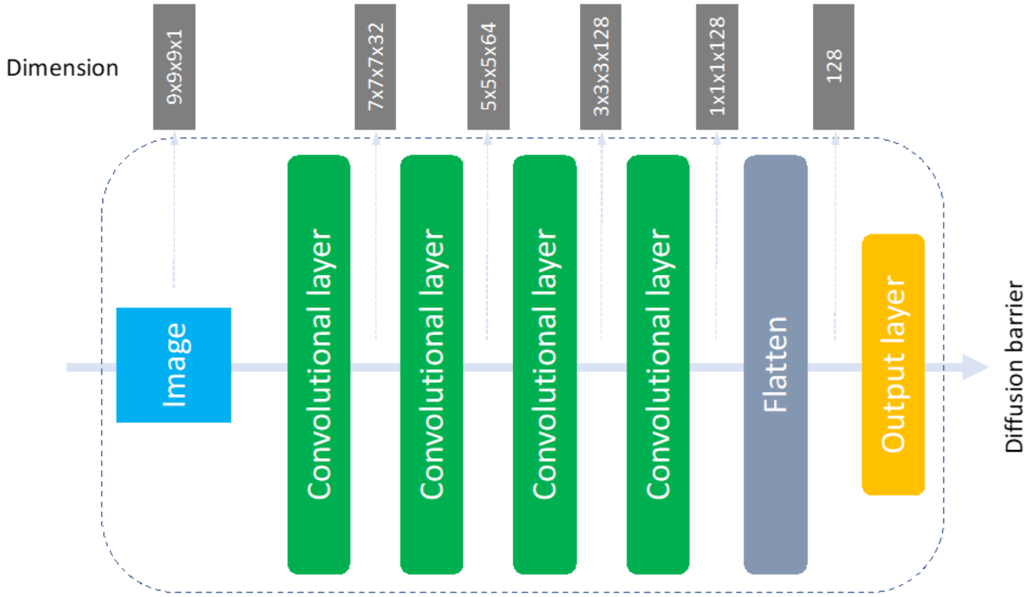

Figure S17. Architecture of the convolutional neural network. The model consists of one input layer, four convolutional layers, and one output layer.

Supplementary Note 4: Number of compositions for predicting the entire ternary composition space

We select different numbers of compositions to train both neutral network and CNN models, in order to understand how many compositions are required to predict the entire compositional space. Figure S18a depicts the 46 compositions uniformly distributed in the compositional space. Figure S18b-d, illustrate 1, 4 and 10 compositions (red colored points) located at the center region of the compositional space, respectively. Each composition comprises 16000 barrier data points. For 1-composition dataset, 80% and 20% data are used for training and validation respectively. For 4-composition dataset, 90% and 10% data are used for training and validation respectively. For 10-composition and 46-composition datasets, 95% and 5% data are used for training and validation, respectively.

Figure S19 presents the prediction performance (i.e., MAEs) in an unseen equimolar NbMoTa alloy as function of number of compositions used for training models. The prediction error decreases rapidly when the number of training compositions increases from 1 to 4. When the number of compositions increases from 4 to 46, the prediction error is further lowered with a small amount. The trend indicates that the addition of data from dilute solutions (i.e., the corners of compositional space) can improve model prediction performance, but not as significant as concentrated solutions. The CNN model performance from 4-composition dataset (MAE = 0.026 eV) is remarkable (the average barrier is around 1.5 eV), which implies that the CNN model has deciphered the chemical complexity and successfully linked it with diffusion barriers. As to the neural network models, it is worthwhile to note that the prediction errors barely change when we increase the number of hidden layers, suggesting the 4 layers of neural network are sufficient for the barrier prediction. Compared to neural network, the CNN shows enhanced performance with lower MAEs, implying the added convolutional layers capture the large-scale atomic patterns contributing to vacancy migration.

It is worth nothing that the testing performance of the trained neural networks is evaluated using newly generated data from other (unseen) compositions, which are not used for training and validation. Figure S20 shows the testing results for these new compositions, $Nb_{10}Mo_{10}Ta_{80}$, $Nb_{20}Mo_{60}Ta_{20}$, $Nb_{40}Mo_{30}Ta_{30}$, and the average MAE is smaller than 0.018 eV, implying the generalizability. Notably the neural networks trained using small configurations precisely predict diffusion barriers in large atomic configuration. For instance, the neural network preserves the consistent high accuracy for different sized systems containing 512, 2000, and 6750 atoms, indicating scalability.

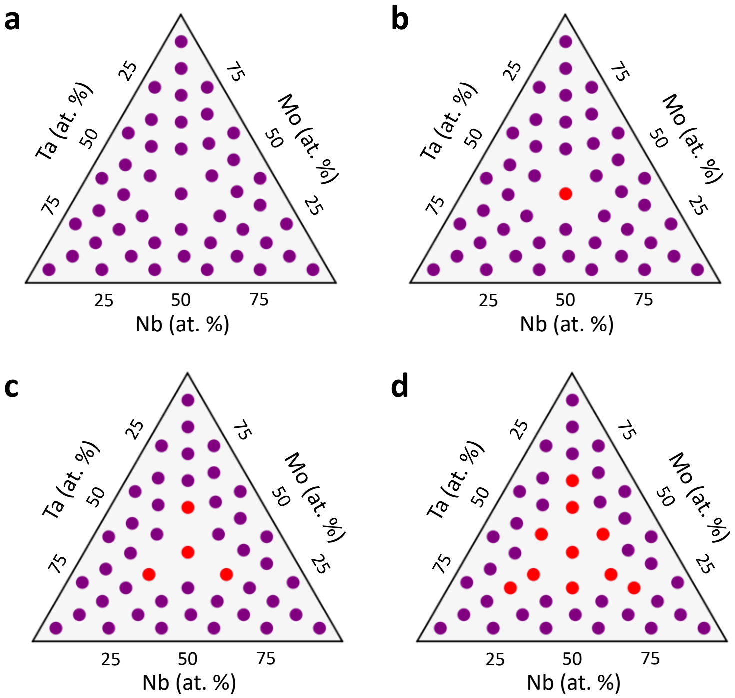

Figure S18. Compositions used for building different training datasets. (a) depicts forty-six compositions occupying the NbMoTa compositional space uniformly. (b-d) depict one composition, four compositions, ten compositions (red-colored points) located at the center of the compositional space representing concentrated alloys.

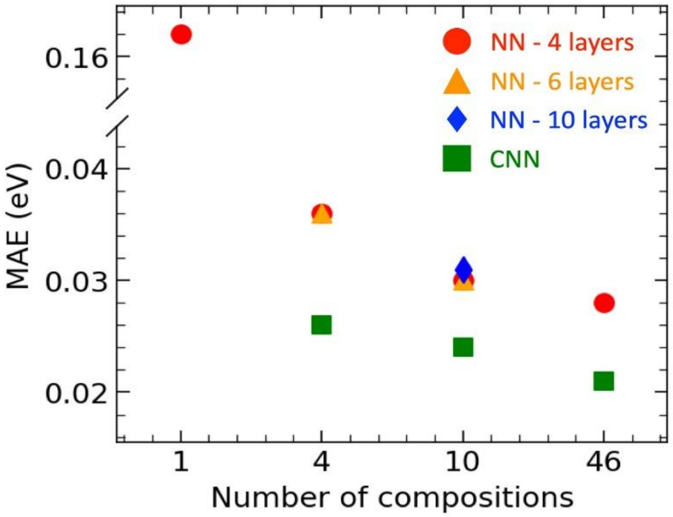

Figure S19. Prediction error of neural network (NN) and CNN as a function of the number of compositions used during training. The evaluation is done on previously unseen compositions in Nb-Mo-Ta, and the results indicate that including more than four compositions leads to a rapid convergence of the network's performance.

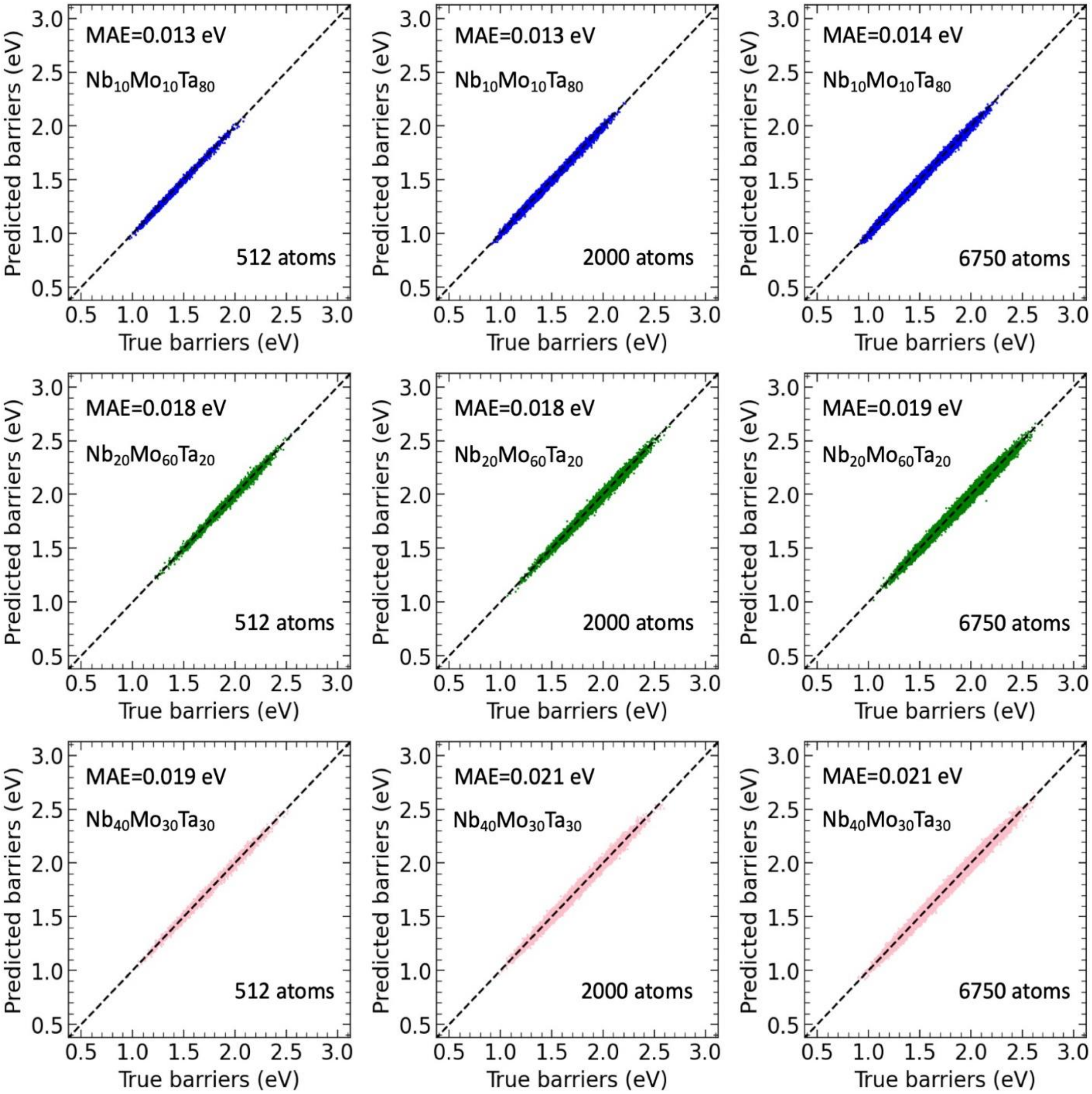

Figure S20. Performance of CNN in predicting diffusion barrier spectrum in unseen compositions and varying system sizes (scalability). Three compositions, including $Nb_{10}Mo_{10}Ta_{80}$, $Nb_{20}Mo_{60}Ta_{20}$, $Nb_{40}Mo_{30}Ta_{30}$, and three systems containing 512, 2000, and 6750 atoms are shown. The architecture of Convolutional neural network is illustrated in Figure S14.

Supplementary Note 5: Comparison with cluster expansion method

The cluster expansion (CE)[1,2] method is often used to predict thermodynamic properties of multicomponent systems, such as vacancy formation energy[3]. The total energy of a configuration is computed by summing up a series of clusters (for instance, single, pair, triplet, and large group of atoms). For kinetics problems such as vacancy diffusion, the governing property is the underlying diffusion activation energy ΔE, i.e., the energy difference (barrier) between transition state and the initial energy minimum. Figure S1 schematically illustrate vacancy diffusion and its corresponding potential energy landscape. The process starts from an initial state E_i, through a transition state E_s, and leads to the neighboring local minimum, i.e., final state E_f. The CE has been adopted to predict the energies of local minimum states. However, addressing the transition state presents specific challenges in CE method.

In the classic application of CE for predicting configurational energy, the initial critical steps involve designing unique clusters based on lattice symmetry and choosing an optimal cluster set, which can be a time-consuming process. For example, the property of vacancy can be influenced by the atoms in its 8th neighboring shell. It has been noted that it can take a number of weeks to months to select optimal clusters when the 8th nearest neighboring atoms is considered [4]. There have been attempts to use CE method for predicting saddle point energy in binary alloys[4], and to our best of knowledge, only one study[5] focusing on predicting vacancy barriers in compositionally complex alloys (ternary alloys). There are notable challenges associated with employing CE for diffusion barrier prediction.

(i) Saddle point representation. To model the transition state (saddle point) for energy prediction using CE, the strategy is to introduce artificial atoms. In a binary system, the jumping species could be either atom type 1 or 2. To differentiate, two additional species, type 3 and type 4, are introduced. This increases chemical complexity and the intricacy of cluster design. In a N-component system, N extra species need to be defined, leading to a total of 2N+1 species (including vacancy).

(ii) CE prediction performance in ternary alloy. To predict vacancy diffusion barrier in ternary system (Al-Mg-Zn), the CE is combined with a quartic function of the reaction coordinate[5]. Comparing with the predicted diffusion barriers with the ground truth obtained from NEB calculation, the mean error of CE is 0.0451 eV, approximately 10% of the average actual barrier. This is contrasted with our model, as shown in Figure 2 of the manuscript, which achieves a significantly lower mean error of 1.2%, an order of magnitude lower than that of CE.

We discuss some key features associated with our neural network kinetics model, which render its highly accurate barrier prediction. It is noted that the model performance in highly accurate barrier prediction is not just for a single alloy composition but across a wide range of varying alloy compositions (the entire compositional space of the ternary alloys).

a. Neuron map representation of atomic structure and chemistry: The neuron map (on-lattice) representation precisely captures atomic structure and composition. Its dimension $O(N)$ scales linearly with the number of atoms N, and has the lowest dimensionality possible as a crystal descriptor. Critically, determining the neuron map is simple and involves simple calculation (avoiding the painstaking parameter tuning in other methods, such as cluster design in CE).

b. Predict performance: The model exhibits high accuracy in barrier prediction. For instance, the mean absolute errors (MAE) for dilute solution $Nb_{90}Mo_5Ta_5$ and concentrated solution $Nb_{33}Mo_{33}Ta_{33}$ are 0.011 and 0.021 eV, respectively. The error is smaller than 1.2% of the true diffusion barrier.

c. Generalization and predicting in entire compositional space: More importantly for compositionally complex materials possessing a vast compositional space, the current method, trained on dozens of compositions, shows remarkable predictability for new (previously unseen) compositions, allowing accurate mapping of the entire ternary space (Figure 2c of manuscript).

d. Scalability and efficiency: Our model demonstrates scalability with system size. This size scalability is shown by accurate barrier predictions in larger NbMoTa systems. For example, the neural network preserves a consistent high accuracy for different sized systems containing 512, 2000, and 6750 atoms.

e. High efficiency in modeling diffusion: neural map originates from its simplicity to mirror vacancy jumps through the swapping of neurons (digits). With only one-time conversion of atomic configuration to neuron map, vacancy jumps and chemical evolution can be simulated by swapping two digits of neural map. In this way, millions of vacancy jumps can be modeled efficiently, with each jump iteration involving the action of just two neurons. Using one single CPU, the model evolves 10 million diffusion jumps in a large system containing 128,000 atoms within two days.

In contrast to the descriptors used in machine learning potentials, our neuron map, representing the entire system, has the smallest possible dimension of $O(N)$. This small dimensionality as a system descriptor, coupled with our neuron kinetics operation, makes modeling vacancy diffusion at this fast speed, surpassing those of classical MD and MC algorithms.

In summary, as compared with traditional CE, our introduced neuron map representation and the computational scheme are more accurate (low error prediction), capable of predicting vacancy barriers in the entire space using small training data (Figure 2c of the manuscript), and efficient and fast (Figure 5, evolving 10 million jumps in large system).

Supplementary Tables

Table S2. Dataset for determining the cutoff distance

Index	Composition	System size (atoms)	Number of barriers (NEB calculation)	Total number of barriers
1	$Nb_{33}Mo_{33}Ta_{33}$	2,000	16,000	64,000
2	$Nb_{50}Mo_{25}Ta_{25}$	2,000	16,000	
3	$Nb_{25}Mo_{50}Ta_{25}$	2,000	16,000	
4	$Nb_{25}Mo_{25}Ta_{50}$	2,000	16,000	

Table S3. Dataset used for predicting vacancy diffusion barriers in the entire Nb-Mo-Ta space

Index	Composition	System size (atoms)	Number of barriers (NEB calculation)	Total number of barriers
1	$Nb_{5}Mo_{5}Ta_{90}$	2,000	16,000	736,000
2	$Nb_{5}Mo_{22}Ta_{73}$	2,000	16,000	
3	$Nb_{5}Mo_{39}Ta_{56}$	2,000	16,000	
4	$Nb_{5}Mo_{56}Ta_{39}$	2,000	16,000	
5	$Nb_{5}Mo_{73}Ta_{22}$	2,000	16,000	
6	$Nb_{10}Mo_{10}Ta_{80}$	2,000	16,000	
7	$Nb_{10}Mo_{27}Ta_{63}$	2,000	16,000	
8	$Nb_{10}Mo_{44}Ta_{46}$	2,000	16,000	
9	$Nb_{10}Mo_{61}Ta_{29}$	2,000	16,000	
10	$Nb_{15}Mo_{15}Ta_{70}$	2,000	16,000	
11	$Nb_{15}Mo_{33}Ta_{52}$	2,000	16,000	
12	$Nb_{15}Mo_{51}Ta_{34}$	2,000	16,000	
13	$Nb_{20}Mo_{20}Ta_{60}$	2,000	16,000	
14	$Nb_{20}Mo_{40}Ta_{40}$	2,000	16,000	
15	$Nb_{5}Mo_{90}Ta_{5}$	2,000	16,000	
16	$Nb_{22}Mo_{73}Ta_{5}$	2,000	16,000	
17	$Nb_{39}Mo_{56}Ta_{5}$	2,000	16,000	
18	$Nb_{56}Mo_{39}Ta_{5}$	2,000	16,000	

19	$Nb_{73}Mo_{22}Ta_5$	2,000	16,000
20	$Nb_{10}Mo_{80}Ta_{10}$	2,000	16,000
21	$Nb_{27}Mo_{63}Ta_{10}$	2,000	16,000
22	$Nb_{44}Mo_{46}Ta_{10}$	2,000	16,000
23	$Nb_{61}Mo_{29}Ta_{10}$	2,000	16,000
24	$Nb_{15}Mo_{70}Ta_{15}$	2,000	16,000
25	$Nb_{33}Mo_{52}Ta_{15}$	2,000	16,000
26	$Nb_{51}Mo_{34}Ta_{15}$	2,000	16,000
27	$Nb_{20}Mo_{60}Ta_{20}$	2,000	16,000
28	$Nb_{40}Mo_{40}Ta_{20}$	2,000	16,000
29	$Nb_{90}Mo_5Ta_5$	2,000	16,000
30	$Nb_{73}Mo_5Ta_{22}$	2,000	16,000
31	$Nb_{56}Mo_5Ta_{39}$	2,000	16,000
32	$Nb_{39}Mo_5Ta_{56}$	2,000	16,000
33	$Nb_{22}Mo_5Ta_{73}$	2,000	16,000
34	$Nb_{80}Mo_{10}Ta_{10}$	2,000	16,000
35	$Nb_{63}Mo_{10}Ta_{27}$	2,000	16,000
36	$Nb_{46}Mo_{10}Ta_{44}$	2,000	16,000
37	$Nb_{29}Mo_{10}Ta_{61}$	2,000	16,000
38	$Nb_{70}Mo_{15}Ta_{15}$	2,000	16,000
39	$Nb_{52}Mo_{15}Ta_{33}$	2,000	16,000
40	$Nb_{34}Mo_{15}Ta_{51}$	2,000	16,000
41	$Nb_{60}Mo_{20}Ta_{20}$	2,000	16,000
42	$Nb_{40}Mo_{20}Ta_{40}$	2,000	16,000
43	$Nb_{50}Mo_{25}Ta_{25}$	2,000	16,000
44	$Nb_{25}Mo_{50}Ta_{25}$	2,000	16,000
45	$Nb_{25}Mo_{25}Ta_{50}$	2,000	16,000
46	$Nb_{33}Mo_{33}Ta_{33}$	2,000	16,000

Supplementary References

1. Mayer, J. E. & Montroll, E. Molecular Distribution. *J Chem Phys* **9**, 2–16 (1941).

2. Laks, D. B., Ferreira, L. G., Froyen, S. & Zunger, A. Efficient cluster expansion for substitutional systems. *Phys Rev B* **46**, 12587–12605 (1992).

3. Van der Ven, A. & Ceder, G. Vacancies in ordered and disordered binary alloys treated with the cluster expansion. *Phys Rev B* **71**, 054102 (2005).

4. Zhang, X. & Sluiter, M. H. F. Cluster Expansions for Thermodynamics and Kinetics of Multicomponent Alloys. *J Phase Equilibria Diffus* **37**, (2016).

5. Xi, Z., Zhang, M., Hector, L. G., Misra, A. & Qi, L. Mechanism of local lattice distortion effects on vacancy migration barriers in fcc alloys. *Phys Rev Mater* **6**, (2022).
