# Supplementary material for: Neural network kinetics for exploring diffusion multiplicity and chemical ordering in compositionally complex materials
Source: Nat Commun. 2024 May 9;15:3879. doi: 10.1038/s41467-024-47927-9 (PMC11082203; doi:10.1038/s41467-024-47927-9)
Supplement: Supplementary file 3 — Description of Additional Supplementary Information [file 41467_2024_47927_MOESM3_ESM.pdf]

Description of Additional Supplementary Information

File Name: Supplementary Video 1
Description: Nucleation and growth of B2 clusters.
